# New species of *Prepseudatrichia* Kelsey from Thailand (Diptera, Scenopinidae)

**DOI:** 10.3897/zookeys.122.1598

**Published:** 2011-08-11

**Authors:** Shaun L. Winterton

**Affiliations:** California State Collection of Arthropods, Plant Pest Diagnostics Center, California Department of Food & Agriculture, Sacramento, California, USA

**Keywords:** Therevoid clade, Asiloidea, Scenopinidae

## Abstract

A new species of *Prepseudatrichia* Kelsey, 1969 (*Prepseudatrichia tiger* **sp. n.**) is described from Thailand, the first record of the genus from the Oriental region. A key to world species of *Prepseudatrichia* is given.

## Introduction

Window flies (Diptera: Scenopinidae) are a small family (*ca.* 420 species in 24 extant genera) of cosmopolitan asiloid flies with an adult body size rarely exceeding 5.0 mm. Scenopinids are distributed throughout all major biogeographical regions, but with significant continental endemism at the genus level, and most genera confined to one or two biogeographical regions (Kelsey, 1973).

The genus *Prepseudatrichia* Kelsey, 1969 contains four described species from Africa (*Prepseudatrichia mateui* Kelsey, 1969, *Prepseudatrichia stenogaster* (Séguy, 1931) and *Prepseudatrichia violacea* Kelsey, 1969) and central Asia (Turkmenistan) (*Prepseudatrichia kelseyi* Krivosheina, 1980). These rare flies are notable for their elongate, glossy black body habitus, similar to members of the genera *Pseudatricha* Osten Sacken, 1877 and *Neopseudatrichia* Kelsey, 1969. The elongate body shape and lack of pile in adults of these genera is presumed to be a morphological adaptation for escaping the narrow confines of wood boring beetle galleries, where species in these genera appear as specialist predators of wood boring beetle larvae. The larval and pupal stages of *Prepseudatrichia kelseyi* were described by Krivosheina (1980). *Prepseudatrichia* is differentiated from all other window fly genera based on the wing vein M1 being fused to the wing margin, separate from R5, an elongate glossy black body, the male genitalia with a well developed hypandrium, an aedeagus extending anteriorly into the body cavity and lateral aedeagal lobes well developed, and the female lacking acanthophorite spines. As mentioned by Kelsey (1969), while *Prepseudatrichia* has a body shape similar to *Pseudatrichia* and *Neopseudatrichia*, the male genitalic morphology and wing venation more closely resemble members of *Scenopinus* Latreille, 1802. This may indicate a closer relationship to *Scenopinus* and the elongate, glabrous adult morphology representing convergence associated with larval feeding in wood boring beetle galleries. This question remains to be tested in a quantitative phylogenetic context.

A distinctive new species of *Prepseudatrichia* (*Prepseudatrichia tiger* sp. n.) is described herein from Thailand based on a single male specimen. This is a new geographical record for this genus, previously known only from the Afrotropical and Palaearctic regions. A key to species of *Prepseudatrichia* is presented.

## Materials and methods

Genitalia were macerated in 10% KOH at room temperature for one day to remove soft tissue, then rinsed in distilled water and dilute acetic acid, and dissected in 80% ethanol. Preparations were then placed into glycerine, with images made with the aid of a digital camera mounted on a stereomicroscope. Genitalia preparations were placed in glycerine in a genitalia vial mounted on the pin beneath the specimen. Terminology follows McAlpine (1981) and modified following Winterton (2005) and Winterton and Woodley (2009). In contrast to the scenopinid subfamilies Proratinae and Caenotinae, the male terminalia of Scenopininae are rotated 180°. To avoid confusion with terminology and comparative homology, structures are described and labeled as they are in related flies with terminalia not rotated; therefore the ventral apodeme of the aedeagus described herein is physically located dorsally. Type material is deposited in the Queen Sirikit Botanic Garden – Entomology collection, Chiang Mai, Thailand (QSBG). Specimen images were taken using a digital camera with a series of images montaged using Helicon Focus (©HeliconSoft).

## Taxonomy

### Key to Prepseudatrichia species

(modified after Kelsey 1969; males are unknown for *Prepseudatrichia violacea* and *Prepseudatrichia stenogaster*; females are unknown for *Prepseudatrichia tiger* sp. n.)

**Table:** 

1	Male	2
–	Female	4
2	Abdominal segments 3 and 4 with white bands; hypandrial lobes extended posteriorly as narrow triangular processes	3
–	Abdomen without white bands; hypandrial lobes truncated, not extended as triangular processes (Turkmenistan)	*Prepseudatrichia kelseyi* Krivosheina
3	Femora brown; hypoproct narrow, extending posteriorly well beyond epandrial lobes (Thailand)	*Prepseudatrichia tiger* sp. n
–	Femora dark yellow; hypoproct truncated, not extending beyond epandrial lobes (North Africa)	*Prepseudatrichia mateui* Kelsey
4	Thorax black, often with metallic luster	5
–	Thorax with green and purple metallic suffusion (Chad)	*Prepseudatrichia violacea* Kelsey
5	Femora black	*Prepseudatrichia kelseyi* Krivosheina
–	Femora with red or yellow suffusion	6
6	Femora reddish; flagellum orange	*Prepseudatrichia stenogaster* (Séguy)
–	Femora yellowish; flagellum black-brown	*Prepseudatrichia mateui* Kelsey

#### Prepseudatrichia tiger sp. n.

urn:lsid:zoobank.org:act:FC2E1669-10A7-487F-8C15-1A1E7577FD2D

http://species-id.net/wiki/Prepseudatrichia_tiger

Figs 1 2 

##### Type material.

**Holotype** male, THAILAND: Loei Phu Ruea National Park, Nern Wibaak ditch, 17°29.907'N, 101°20.483'E, 1196m, Malaise trap 26.ii-2.iii.2007, Patikhom Tumtip leg. T1714 (QSBG).

**Figure 1.**
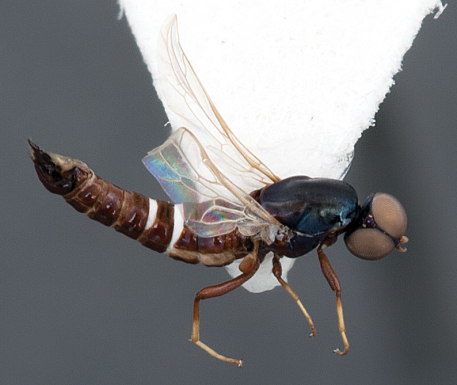
*Prepseudatrichia tiger* sp. n.: Male holotype habitus. Body length= 4.1 mm.

##### Diagnosis.

 Male abdomen with white bands on segments 3–4; antenna yellow-brown; thorax glossy black with metallic iridescence; femora brown; hypandrial lobes with triangular processes extending posteriorly to apex on epandrial lobes; hypoproct narrow, extending beyond epandrial lobes.

##### Description.

**Figure 2.**
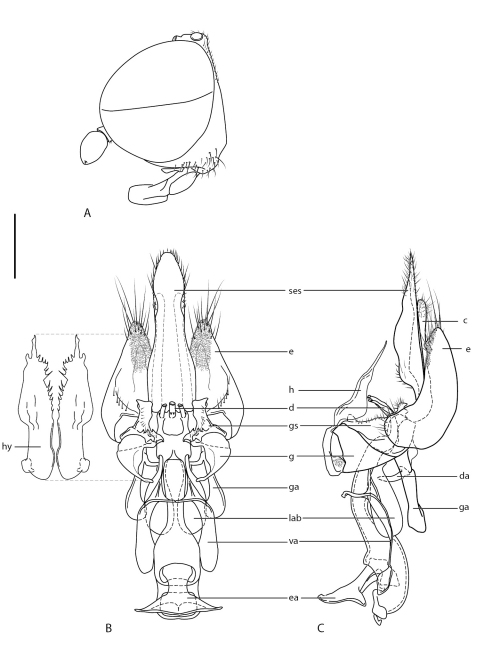
*Prepseudatrichia tiger* sp. n. **A** Male head, lateral view. Male genitalia **B** dorsal view (physically dorsal), hypandrium removed to show internal structures **C** same lateral view with hypandrium in place. Scale line = 0.25 mm. Abbreviations: *c*, cercus; *d*, distiphallus; *da*, dorsal apodeme of parameral sheath; *e*, epandrium; *ea*, ejaculatory apodeme; *g*, gonocoxite; *ga*, gonocoxal apodeme; *gs*, gonostylus; *h*, hypandrium; *ses*, subepandrial sclerite; *lab*, lateral aedeagal bulb; *va*, ventral apodeme of parameral sheath.

 Male. Body length: 4.1 mm (female unknown). *Head*. Glossy brown-black (Fig. 1); frons not protruding anteriorly beyond eye in profile (Fig. 2a), eyes almost contiguous at narrowest point; face brown-black, parafacia narrow, glabrous; mouthparts brown; antenna yellow-brown, overlain with greyish pubescence, admixed with few short setae on outer surface of scape and pedicel, style subterminal; ocellar tubercle raised, broad; postocular ridge very narrow, with few minute setae laterally; occiput relatively flat to concave, pale pubescent medially; gena sparsely covered with fine yellowish setae. *Thorax*. Glossy brown-black with metallic iridescence, scutum finely rugose to scrobiculate posteromedially, very sparsely overlain with short pale setae; postpronotal lobe and postalar ridge pale tan; pleuron smooth and polished, except for a few sparse fine setae; coxae and femora brown; tibia brown basally, dark-yellow apically; sparse, short setae on legs; haltere dark brown; wing hyaline; venation typical for genus, dark yellow. *Abdomen*. Glossy brown, cylindrical, glabrous; dorsal surface flattened with dark, alveolate texture resembling elongate honeycomb; intersegmental membranes and posterior margins of tergites 3–4 bright white; tergite 2 sensory setal region as two circular patches. *Male genitalia* (Fig. 2B–C). Epandrial lobes scoop-like, not enclosing gonocoxites, elongate setae apically, microtrichia posteromedially; cerci narrow; hypoproct relatively large, narrow, extending beyond cerci posteriorly; gonocoxite triangular in shape; gonostylus well developed with spinose ridge posteriorly, articulated on gonocoxite basally; gonocoxal apodeme relatively narrow; hypandrial lobes membranous, elongate with irregular posteromedial margin, lobes articulated ventrally on gonocoxites; aedeagus extended anteriorly well beyond genitalic capsule; distiphallus trifid, curved, not extending beyond genitalic capsule; lateral aedeagal bulbs well developed either side of basiphallus; ventral apodeme of parameral sheath greatly enlarged, arms flanking aedeagus anteriorly with brace-like flanges; dorsal apodeme small, dark sclerotized as brace between gonocoxal apodemes; ejaculatory apodeme relatively large spatulate, directed ventrally from bulbous basiphallus.

##### Etymology.

The species epithet is derived from the acronym for the Thailand Inventory Group for Entomological Research (TIGER) project, from which this species was discovered.

##### Comments.

 This species is known only from a single male specimen collected in Thailand. This represents a considerable range extension for the genus, into the Oriental Region, as *Prepseudatrichia* was previously only known from few species in the Palaearctic and Afrotropical regions. This distinctive species is differentiated from other species in the genus by the shape of the male genitalia, the white bands on the abdomen and dark femora color.

## Supplementary Material

XML Treatment for Prepseudatrichia tiger
